# Etiological analysis and predictive diagnostic model building of community-acquired pneumonia in adult outpatients in Beijing, China

**DOI:** 10.1186/1471-2334-13-309

**Published:** 2013-07-09

**Authors:** Ya-Fen Liu, Yan Gao, Mei-Fang Chen, Bin Cao, Xiao-Hua Yang, Lai Wei

**Affiliations:** 1 Peking University People’s Hospital, Department of Infectious Disease, Peking University Hepatology Institute, No. 11, Xizhimen South Street, Beijing 100044, P. R. China; 2 Department of Infectious Diseases and Clinical Microbiology, Beijing Chao-Yang Hospital, Capital Medical University, No. 8, Gongti South Road, Chaoyang District, Beijing 100020, P. R. China

**Keywords:** Community-acquired pneumonia, Etiology, Epidemiology, Diagnosis, Pneumonia, Virus, Polymerase chain reaction, ROC curve

## Abstract

**Background:**

Etiological epidemiology and diagnosis are important issues in adult community-acquired pneumonia (CAP), and identifying pathogens based on patient clinical features is especially a challenge. CAP-associated main pathogens in adults include viruses as well as bacteria. However, large-scale epidemiological investigations of adult viral CAP in China are still lacking. In this study, we analyzed the etiology of adult CAP in Beijing, China and constructed diagnostic models based on combinations of patient clinical factors.

**Methods:**

A multicenter cohort was established with 500 adult CAP outpatients enrolled in Beijing between November 2010 to October 2011. Multiplex and quantitative real-time fluorescence PCR were used to detect 15 respiratory viruses and mycoplasma pneumoniae, respectively. Bacteria were detected with culture and enzyme immunoassay of the *Streptococcus pneumoniae* urinary antigen. Univariate analysis, multivariate analysis, discriminatory analysis and Receiver Operating Characteristic (ROC) curves were used to build predictive models for etiological diagnosis of adult CAP.

**Results:**

Pathogens were detected in 54.2% (271/500) of study patients. Viruses accounted for 36.4% (182/500), mycoplasma pneumoniae for 18.0% (90/500) and bacteria for 14.4% (72/500) of the cases. In 182 of the patients with viruses, 219 virus strains were detected, including 166 single and 53 mixed viral infections. Influenza A virus represented the greatest proportion with 42.0% (92/219) and 9.1% (20/219) in single and mixed viral infections, respectively. Factors selected for the predictive etiological diagnostic model of viral CAP included cough, dyspnea, absence of chest pain and white blood cell count (4.0-10.0) × 10^9^/L, and those of mycoplasma pneumoniae CAP were being younger than 45 years old and the absence of a coexisting disease. However, these models showed low accuracy levels for etiological diagnosis (areas under ROC curve for virus and mycoplasma pneumoniae were both 0.61, *P* < 0.05).

**Conclusions:**

Greater consideration should be given to viral and mycoplasma pneumoniae infections in adult CAP outpatients. While predictive etiological diagnostic models of viral and mycoplasma pneumoniae based on combinations of demographic and clinical factors may provide indications of etiology, diagnostic confirmation of CAP remains dependent on laboratory pathogen test results.

## Background

Community-acquired pneumonia (CAP) has a major impact on public health and results in more than 10 million visits to physicians and 600,000 hospitalizations each year in the USA [1], where it is the seventh leading cause of death, and its economic burden has been estimated to be more than $17 billion annually [2,3]. Etiological epidemiology and diagnosis are important issues in adult CAP, with particular challenges in identifying the causative pathogens based on patient clinical features. Studies conducted prior to the year 2000 showed that the predominant pathogen of CAP was *Streptococcus pneumoniae*, which accounted for approximately two-thirds of the detected CAP pathogens [4-6]. Later, atypical pathogens began showing increasing trends among CAP cases [7]. In the last two decades, with the rapid development of molecular diagnostic techniques and the outbreak of severe acute respiratory syndrome (SARS) coronavirus, avian influenza A (H5N1) virus and the 2009 pandemic influenza A (H1N1) virus, viral CAP has garnered more attention. Shin [8] pointed out that if clinicians do not consider a viral etiology in patients with CAP, they would be unlikely to consider investigations to diagnose respiratory viruses, which would result in the inappropriate use of antibiotics, missed opportunity to consider antiviral treatment and failure to institute appropriate infection control measures. Ruuskanen *et al.*[9] also stressed the importance of viral CAP and the urgent need to gather epidemiological data on etiological pathogens from developing countries. At present, China still lacks large-scale epidemiological investigations of adult viral CAP. Furthermore, antibiotic resistance is a serious issue in China, as 24.9% of the *S. pneumoniae* has been found to be resistant to penicillin and 87.5% to macrolide [10], while the macrolide resistance rate of mycoplasma pneumoniae (*M. pneumoniae*, MP) in adults has been estimated at 69% [11]. Accordingly, underestimation of the prevalence of viral CAP and utilization of inappropriate treatment may increase the spread of antibiotic resistance.

Based on the considerations above, we investigated the etiology of adult CAP in Beijing, China from November 2010 to October 2011. Furthermore, combinations of clinical factors of this population were analyzed in order to build etiological diagnostic models to predict the viral or *M. pneumoniae* sources of infection.

## Methods

### Study patients

Five hundred adult outpatients were enrolled between November 2010 to October 2011 from 12 hospitals, including 8 teaching hospitals and 4 secondary hospitals, in Beijing, China. These 12 hospitals serve approximately 20 million outpatients annually. Our study subjects were from the outpatient department of infectious diseases or respiratory diseases of these hospitals, at which at least 20,000 CAP patients are seen annually.

The inclusion criteria were based on the following: 1) patients were at least 18 years old; 2) site-of-care decisions for outpatients were made according to the Infectious Diseases Society of America/American Thoracic Society (IDSA/ATS) guidelines on the management of CAP in adults in 2007 [12]; 3) CAP was defined by a new infiltrate on a chest X-ray examined by two radiologists and the presence of one of the following clinical characteristics: new cough or aggravated cough with or without sputum production; fever (> 37.8°C) or hypothermia (< 35.6°C), leukocytosis (> 10 × 10^9^/L) or leukopenia (< 4 × 10^9^/L) [13]; 4) patients agreed to participate in this investigation and accepted the laboratory tests and etiological examination voluntarily. Patients were excluded if they were HIV infected or under an immunosuppressed state, had clinical symptoms for more than 1 week from the time of onset, were pregnant or in the lactation period, were hospitalized within the prior 90 days (hospital stay longer than 2 days), lived in a nursing home or rehabilitation hospital, or had been previously treated with antivirals.

### Data collection

Data on demographic factors (sex, age, smoking, coexisting disease and antibiotic pretreatment), clinical symptoms and signs (fever, body temperature, max temperature, heart rate, respiratory rate, systolic pressure ≤ 90 mmHg, cough, expectoration, dyspnea, chest pain, diarrhea, vomiting, dizziness, headache, moist rales and dry rales) and laboratory test results [white blood cell (WBC) counts, neutrophil, lymphocyte, hematocrit, platelets, alanine aminotransferase, aspartate aminotransferase, blood creatinine, blood sodium, pH, PaO_2_, erythrocyte sedimentation rate, C-reactive protein, prothrombin time and activated partial thromboplastin time] were collected using data abstraction forms for patients meeting the inclusion criteria. This process was accompanied by stringent quality controls, including training specific doctors for recording of information; selecting study patients strictly in accordance with inclusion and exclusion criteria; using standardized abstraction forms to guide data collection in case of conflicting, ambiguous and missing information; defining the coexisting disease, including tumor, coronary heart disease, heart failure, cerebrovascular disease, chronic nephropathy, chronic hepatopathy, diabetes mellitus, CAP hospitalization within the prior one year, chronic obstructive pulmonary and autoimmune disease based on specialist opinion; ensuring laboratory tests were obtained from a nationally accredited laboratory; and establishing a monitoring system for the collection of information.

### Sample collection

A single throat swab, using Sterile Rayon Swabs (167KS01, Guangzhou, China), was collected from each study patient before receiving antivirals or antibiotic drugs. The swab was immediately placed in a virus transport media tube (167KS01, Guangzhou, China). Each sample was frozen at −80°C within 24 h until analyzed.

Sputum or blood was obtained for bacterial culture before antibiotic therapy, while blood culture was usually performed when the patient temperature was higher than 38.5°C. Patients were instructed to produce a deep expectoration after gargle into a sterile, dry, impermeable, non-absorbent container within 2 h before the test. Ten milliliters of blood was obtained and inoculated into two culture bottles (5 mL was inoculated into an aerobic bottle and 5 mL into an anaerobic bottle). Thirty minutes later, an additional 10 mL of blood was obtained from a different site, and the procedure was repeated. We obtained altogether 369 sputum samples and 85 sets of blood samples. Middle clean urine samples for *S. pneumoniae* testing were obtained 1 day after enrollment for all study patients.

### Detection of viruses and *M. pneumoniae*

Multiplex PCR (Neuro-Hemin Biotech Co., Ltd, Hangzhou, China) was used to detect 15 common respiratory viruses by throat swabs, including influenza A virus (Flu A) , influenza B virus (Flu B), parainfluenza virus types 1, 2, 3, and 4 (PIV1, 2, 3, 4), respiratory syncytial virus types A and B (RSVA and RSVB), adenovirus (AdV), human coronavirus 229E/NL63, OC43 (CoV 229E/NL63, OC43), rhinovirus HRV A/B/C (hRV), human bocavirus 1/2/3/4 (hBoV), human metapneumovirus (hMPV) and enterovirus (EnV). Quantitative real-time fluorescence PCR was used for detection of *M. pneumoniae* in throat swabs. Detections of viruses and *M. pneumoniae* were performed in the Clinical Microbiology Department in the Beijing Chao-Yang Hospital of Capital Medical University.

DNA and RNA were extracted from samples using the QIAamp DNA Mini kit (Cat. No.51306, Qiagen, Hilden, Germany) and QIAamp Viral RNA Mini Kit (Cat. No.52906, Qiagen), following the manufacturer’s instructions. The extracted RNA was used as template to perform the reverse transcriptase polymerase chain reaction (RT-PCR) with a commercial kit (Fermentas, Shenzhen, China) as follows. Total RNA (8 μl), random hexamers (1 μl of 0.2 μg/μl) and DEPC-treated water (3 μl) were added to an RT tube on ice, incubated at 80°C for 3 min and then chilled on ice again for 2 min. Thereafter, 5× RT buffer (4 μl), 10 mm dNTP (2 μl), RNase inhibitor (1 μl of 20 U/μl) and reverse transcriptase (1 μl of 200 U/μl) were added to the tube, which was then incubated at 37°C for 90 min, followed by 94°C for 2 min. After chilling on ice for a further 2 min, the complementary DNA (cDNA) generated from the reverse transcription was stored at −20°C until ready for use. The PCR amplification system included the cDNA template (3 μl), 5× RV Primer (4 μl), 8-Mop Solution (3 μl) and 2× Multiplex Master Mix (10 μl), and the conditions were: 94°C for 15 min, followed by 40cycles of 94°C for 0.5 min, 60°C for 1.5 min and 72°C for 1.5 min, with extension at 72°C for 10 min. The product was stored at 4°C until used. All PCR products, markers and negative control were analyzed by electrophoresis in 1% agarose gels stained with ethidium bromide and visualized using the Molecular Imager Gel Doc XR System (Bio-Rad 170–8170, Hercules, CA, USA). The Quantitative Diagnosis Kit for *M. pneumoniae* DNA (PCR Fluorescence Probing, Da An Co., Ltd, Guangzhou, China) was used for *M. pneumoniae* detection with the following PCR amplification system: DNA template (2 μl), MP PCR reaction mix (40 μl) and Taq enzyme (3 μl). The PCR reaction was carried out using a quantitative PCR instrument (ABI Prism 7500, USA) with the following conditions: 93°C for 2 min, followed by 10 cycles of 93°C for 45 sec and 55°C for 1 min, and another 30 cycles of 93°C for 0.5 min and 55°C for 10 min.

### Detection of bacteria

All sputum samples were examined by microscopy, and representative sputum originating from the lower respiratory tract was defined as that containing > 25 granulocytes and < 10 epithelial cells per field of view under a low power microscope. The standard four zoning line method was used for semi-quantitative culture. Conventional blood culture was processed in an automated system. *S. pneumoniae* urine antigen was detected using an enzyme immunoassay. Detection of bacteria was performed at each hospital laboratory.

### Statistical analysis

Statistical analysis was performed using SPSS statistical software version 16.0 (SPSS Inc., Chicago, IL, USA). Discrete variables were expressed as counts (percentage) and continuous variables as means ± SD or median (interquartile range). Frequency comparisons were made with the chi-square test. Two-group comparisons of normally distributed data were performed with the independent samples t-test. For multi-group comparisons, one-way analysis of variance (ANOVA) with the least-squares difference *post hoc* test was applied. For data not normally distributed, the Mann–Whitney U test was used if only two groups were compared, and Kruskal-Wallis one-way ANOVA was used if more than two groups were being compared. Variables with *P* values less than 0.2 in univariate analysis were used in multivariate logistic regression analysis. The removal probability for multivariate stepwise logistic regression analysis was 0.1. Discriminatory analysis and Receiver Operating Characteristic (ROC) curves were used to build and assess the predictive etiological diagnostic models. A probability of *P* < 0.05 was considered to be statistically significant. For convenience in the analysis, the following pathogens were grouped as follows: CoV 229E/NL63, CoV OC43 (CoV); PIV1, 2, 3, 4 (PIV); RSVA and RSVB (RSV).

### Ethics statement

The study protocol was in accordance with the Declaration of Helsinki and was approved by the ethics committees of the 12 participating hospitals (Peking University People’s Hospital, Beijing Chao-Yang Hospital, Beijing Haidian Hospital, YanTai Yu Huangding Hospital, Luhe Teaching Hospital of the Capital Medical University, WangJing Hospital of China Academy of Chinese Medical Sciences, Peking Union Medical College Hospital, China-Japan Friendship Hospital, Beijing Friendship Hospital, Beijing Pinggu Hospital, Huairou the First Hospital and Air Force General Hospital, PLA) in the study. The nature, purpose and potential risks of this study were carefully explained to each subject prior to enrollment. Written informed consent was obtained from all enrolled patients.

## Results

### Pathogen distribution

**Figure 1:**
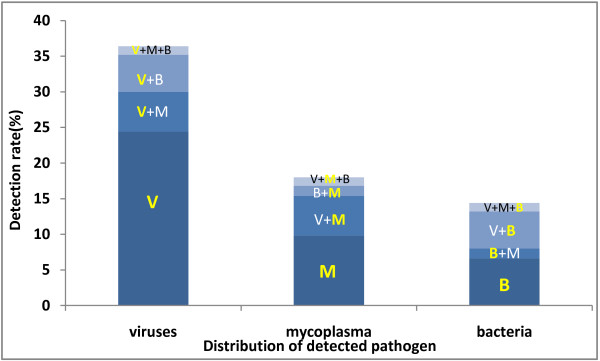
**Pathogen distribution.** (V, virus; M, mycoplasma pneumoniae; B, bacteria; +, mixed).

Pathogens were detected in 54.2% (271/500) of study patients, and the mixed infection ratio of different pathogens was 13.4% (67/500). In our study, viruses accounted for 36.4% (182/500), *M. pneumoniae* for 18.0% (90/500) and bacteria for only 14.4% (72/500) of the cases (Figure 1). Based on the detected pathogens, the 500 enrolled patients were divided into five groups as shown in Tables 1, 2, and 3.

**Table 1:** Demographic factors of patients in different groups

	**Groups**					
	**V(n = 122)**	**M(n = 49)**	**B(n = 33)**	**MIX(n = 67)**	**N(n = 229)**	***P***
Male sex (%)	74(60.7)	26(53.1)	25(75.8)	45(67.2)	120(52.4)	>0.05
Age (years)	43(18–94)	32(18–75)	48(18–84)	33(18–80)	47(18–86)	<0.05
18-44	63(51.6)	38(77.6)	14(42.4)	47(70.1)	110(48.0)	>0.05
45-59	25(20.5)	6(12.2)	11(33.3)	6(9.0)	46(20.0)	>0.05
≥60	34(27.9)	5(10.2)	8(24.2)	14(20.9)	73(31.9)	>0.05
Smoking (%)	30(24.6)	10(20.4)	13(39.4)	13(19.4)	56(24.5)	>0.05
Coexisting disease (%)	17(13.9)	5(10.2)	4(12.1)	5(7.5)	47(20.5)	>0.05
Tumor (%)	3(2.5)	1(2.0)	0(0.0)	0(0.0)	3(1.3)	>0.05
Coronary heart disease (%)	7(5.7)	3(6.1)	3(9.1)	2(3.0)	20(8.7)	>0.05
Cardiac insufficiency (%)	2(1.6)	0(0.0)	0(0.0)	0(0.0)	5(2.2)	>0.05
Cerebrovascular disease (%)	5(4.1)	1(2.0)	2(6.1)	2(3.0)	11(4.8)	>0.05
Chronic nephropathy (%)	1(0.8)	1(2.0)	0(0.0)	0(0.0)	6(2.6)	>0.05
Chronic hepatopathy (%)	1(0.8)	0(0.0)	0(0.0)	0(0.0)	3(1.3)	>0.05
Diabetes mellitus (%)	4(3.2)	2(4.1)	1(3.0)	2(3.0)	21(9.2)	>0.05
CAP hospitalization within 1year (%)	0(0.0)	0(0.0)	0(0.0)	0(0.0)	2(0.9)	>0.05
COPD^*^ (%)	4(3.2)	0(0.0)	0(0.0)	1(1.5)	3(1.3)	>0.05
Autoimmune disease (%)	1(0.8)	0(0.0)	0(0.0)	0(0.0)	0(0.0)	>0.05
Antibiotic pretreatment (%)	75(61.5)	35(71.4)	24(72.7)	42(62.7)	147(64.1)	>0.05

Notes: V: single viral infection; M: single mycoplasma pneumoniae infection; B: single bacterial infection; MIX: mixed infections; N: no pathogen detected.*COPD, chronic obstructive pulmonary disease.

**Table 2:** Symptoms and clinical signs of patients in different groups

	**Groups**					
	**V(n = 122)**	**M(n = 49)**	**B(n = 33)**	**MIX(n = 67)**	**N(n = 229)**	***P***
Fever (%)	110(90.2)	48(98.0)	31(94.0)	60(89.6)	198(86.5)	>0.05
Body temperature (°C)	37.6 ± 0.9	37.7 ± 1.0	37.6 ± 1.1	37.7 ± 1.0	37.6 ± 0.9	>0.05
Max temperature (°C)	38.9 ± 0.7	39.2 ± 0.6	39.2 ± 0.8	39.0 ± 0.8	38.8 ± 2.2	>0.05
Heart rate (beats/minute)	85.9 ± 13.8	91.5 ± 15.3	86.4 ± 13.0	88.4 ± 14.5	85.8 ± 12.3	>0.05
Respiratory rate (breaths/minute)	19.8 ± 2.4	19.5 ± 2.3	20.2 ± 3.5	19.7 ± 2.0	19.9 ± 2.7	>0.05
Systolic pressure ≤ 90 mmHg (%)	0(0.0)	0(0.0)	0(0.0)	0(0.0)	1(0.4)	>0.05
Cough (%)	117(96.0)	46(93.8)	33(100.0)	66(98.5)	208(90.8)	>0.05
Expectoration (%)	89(73.0)	34(69.4)	31(93.9)	61(91.0)	154(67.2)	<0.05
Dyspnea (%)	29(23.8)	6(12.2)	2(6.1)	10(14.9)	32(13.9)	<0.05
Chest pain (%)	13(10.7)	9(18.4)	3(9.1)	2(3.0)	29(12.7)	>0.05
Diarrhea and (or) vomiting (%)	10(8.2)	9(18.4)	4(12.1)	4(6.0)	18(7.9)	>0.05
Dizziness and (or) headache (%)	20(16.4)	9(18.4)	4(12.1)	8(12.0)	36(15.7)	>0.05
Moist rales (%)	46(37.7)	21(42.9)	20(60.6)	31(46.3)	90(39.3)	>0.05
Dry rales (%)	13(10.6)	5(10.2)	3(9.1)	11(16.4)	18(7.9)	>0.05

Notes: V: single viral infection; M: single mycoplasma pneumoniae infection; B: single bacterial infection; MIX: mixed infections.

**Table 3:** Laboratory test results of patients in different groups

	**Groups**					
	**V(n = 122)**	**M(n = 49)**	**B(n = 33)**	**MIX(n = 67)**	**N(n = 229)**	***P***
WBC counts (×10^9^/L)	7.9 ± 3.5	8.4 ± 3.7	7.5 ± 3.4	7.5 ± 3.5	8.7 ± 4.1	>0.05
<4.0 (%)	7(5.7)	5(10.2)	4(12.1)	6(9.0)	12(5.2)	>0.05
4.0 ~ 10.0 (%)	90(73.8)	31(63.3)	24(72.7)	51(76.1)	151(65.9)	>0.05
>10.0 (%)	25(20.5)	13(26.5)	5(15.2)	10(14.9)	66(28.8)	>0.05
Neutrophil (%)	70.6 ± 12.2	70.1 ± 11.0	71.3 ± 13.5	69.0 ± 13.9	69.7 ± 14.5	>0.05
Lymphocyte (%)	20.3 ± 9.8	20.1 ± 8.3	20.5 ± 11.2	21.2 ± 9.9	21.3 ± 11.6	>0.05
Hematocrit (%)	40.9 ± 6.7	39.9 ± 3.9	40.5 ± 5.1	39.3 ± 4.7	40.3 ± 21.7	>0.05
Platelets (×10^9^/L)	209.7 ± 76.8	206.6 ± 65.4	194.6 ± 80.4	202.7 ± 68.0	218.3 ± 66.9	>0.05
Alanine aminotransferase (U/L)	28 ± 22.8	25.0 ± 15.0	45.1 ± 57.0	25.9 ± 20.6	29.3 ± 31.0	>0.05
Aspartate aminotransferase (U/L)	28.3 ± 16.5	26.1 ± 13.1	37.7 ± 37.4	29.4 ± 48.6	26.5 ± 20.1	>0.05
Blood creatinine (μmol/L)	68.7 ± 22.9	64.3 ± 19.5	65.7 ± 14.8	59.8 ± 17.2	66.3 ± 48.3	>0.05
Blood sodium (mmol/L)	137.6 ± 3.7	137.5 ± 4.2	137.8 ± 3	138.0 ± 3.3	138.5 ± 3.4	>0.05
pH	7.4 ± 0.0	7.4 ± 0.0	7.4 ± 0.0	7.4 ± 0.0	7.4 ± 0.1	>0.05
PaO_2_ (mmHg)	80.3 ± 14.9	79.5 ± 17.6	85.5 ± 50.1	85.2 ± 17.8	82.8 ± 19.8	>0.05
ESR^1^ (mm)	34.7 ± 26.1	39.7 ± 21.6	43.5 ± 26.4	39.2 ± 23.9	40.9 ± 28.4	>0.05
C-reactive protein (mg/L)	50.7 ± 60	52 ± 42.8	67.5 ± 56.6	58.8 ± 92.2	62.9 ± 61.7	>0.05
Prothrombin time (s)	12.3 ± 2.2	12.5 ± 1.3	12.7 ± 1.7	12.3 ± 1.6	12.7 ± 3.7	>0.05
APTT^2^(s)	35.8 ± 9.3	35.7 ± 9.5	29.7 ± 6.7	33.1 ± 14.3	32.8 ± 5.7	>0.05

Notes: V: single viral infection; M: single mycoplasma pneumoniae infection; B: single bacterial infection; MIX: mixed infections; N: no pathogen detected. ^1^ESR, erythrocyte sedimentation rate; ^2^APTT, activated partial thromboplastin time.

### Distribution of detected virus

We detected 219 virus strains in the 182 patients with viruses, including 166 single and 53 mixed viral infections. Among the single and mixed viral infections, the respective percentages of each virus were as follows: Flu A, 42.0% (92/219) and 9.1% (20/219); hRV, 9.6% (21/219) and 1.8% (4/219); AdV, 9.1% (20/219) and 2.3% (5/219); PIV, 7.8% (17/219) and 6.8% (15/219); hMPV, 3.2% (7/219) and 1.4% (3/219); RSV, 1.8% (4/219) and 1.4% (3/219); EnV, 1.4% (3/219) and 0%; CoV, 0.9% (2/219) and 0.9% (2/219); Flu B, 0% and 0.5% (1/219).

### Monthly distribution of detected viruses

Flu A infections increased gradually in November 2010, peaked in January 2011 and declined by March 2011 in China. RSV also emerged in November 2010 but could not be detected from February 2011. AdV and hMPV were both detected from January 2011, peaking in February and March, respectively. Infections of hMPV lasted about four months and that of AdV six months. The seasonal distributions of PIV and hRV were not significantly different.

### Patient demographic and clinical factors

We collected data from the 500 patients, including demographic characteristics, symptoms, signs and laboratory test results, as shown in Tables 1, 2, and 3. By comparing symptoms, physical examinations and laboratory test results of monomicrobial infections, we found that CAP patients infected with *M. pneumoniae* were significantly younger than those with viruses or bacteria [*M. pneumoniae* CAP (years of age and age range): 32 (18–75); viral CAP: 43 (18–94); bacterial CAP: 48 (18–84), *P* < 0.05]. Expectoration was more common in bacterial CAP patients compared with the other two groups [bacterial CAP: 93.9% (31/33), viral CAP 73.0% (89/122), *M. pneumoniae* CAP: 69.4% (34/49), *P* < 0.05]. Dyspnea was significantly different between viral and bacterial CAP [23.8% (29/122) vs. 6.1% (2/33), *P* < 0.05] but not in the comparison between *M. pneumoniae* and bacteria [12.2% (6/49) vs. 6.1% (2/33), *P* > 0.05], or *M. pneumoniae* and virus [12.2% (6/49) vs. 23.8% (29/122), *P* > 0.05]. Other characteristics were not statistically significantly different.

### Predictive diagnostic model of viral CAP

We performed univariate logistic analysis of all variables in Tables 1, 2, and 3 for viral CAP and selected the following variables with *P* < 0.2 for multivariate logistic regression analysis: coexisting disease, cough, expectoration, dyspnea, chest pain, dry rales and WBC counts (Table 4). The multivariate logistic analysis identified four independent factors with *P* < 0.1 associated with viral CAP, namely cough (OR 2.40, *P* < 0.1), dyspnea (OR 2.20, *P* < 0.05), chest pain (OR 0.54, *P* < 0.1) and WBC counts (OR 0.93, *P* < 0.05) (Table 5). Subsequently, discriminatory analysis and ROC curves were used to establish and assess predictive diagnostic models of CAP by using these four independent factors. The predictive diagnostic model of viral CAP included cough, dyspnea, absence of chest pain and WBC count (4.0-10.0) × 10^9^/L, with an area under the ROC curve (AUC) of 0.61 (95% CI: 0.55 to 0.68). The sensitivity and specificity of this model were 37.4% (95% CI: 29.1% to 45.7%) and 77.2% (95% CI: 71.5% to 82.9%), respectively (*P* < 0.05).

**Table 4:** **Univariate analysis of clinical factors of patients with viral and mycoplasma pneumoniae CAP (*P*** **< 0.2)**

**Variables**	**Odds ratio**	**(95% CI)**		***P***
Viral CAP				
Coexisting disease	0.64	0.38	1.09	0.103
Cough	2.87	1.08	7.67	0.035
Expectoration	1.47	0.95	2.25	0.081
Dyspnea	1.94	1.19	3.15	0.008
Chest pain	0.61	0.32	1.13	0.113
Dry rales	1.54	0.85	2.77	0.152
WBC counts	0.95	0.91	1.00	0.067
Mycoplasma pneumoniae CAP				
Age	0.96	0.95	0.98	<0.001
Coexisting disease	0.34	0.14	0.80	0.013
Smoking	0.60	0.34	1.08	0.089
Fever	2.70	0.95	7.71	0.063
Max temperature	1.58	1.13	2.20	0.007
Neutrophil	0.99	0.97	1.00	0.121
C-reactive protein	1.00	0.99	1.00	0.078

**Table 5:** **Predictive diagnostic model factors of viral and mycoplasma pneumoniae CAP (*P*** **< 0.1)**

**Variables**	**Odds ratio**	**(95% CI)**		***P***
Viral CAP				
Cough	2.40	0.89	6.52	0.085
Dyspnea	2.20	1.31	3.68	0.003
Chestpain	0.54	0.28	1.03	0.061
WBC counts	0.93	0.88	0.99	0.015
Mycoplasma pneumoniae CAP				
Age	0.94	0.92	0.97	<0.001
Coexisting disease	0.33	0.09	1.21	0.094

### Predictive dagnostic model of *M. pneumoniae* CAP

Univariate logistic analysis was performed with all variables in Tables 1, 2, and 3 for *M. pneumoniae* CAP, and the following variables with *P* < 0.2 were selected for multivariate logistic regression analysis: age, coexisting disease, smoking, fever, max temperature, neutrophil and C-reactive protein (Table 4). Through the multivariate logistic analysis, two independent factors with *P* < 0.1 associated with *M. pneumoniae* CAP were identified, namely age (OR 0.94, *P* < 0.05) and coexisting disease (OR 0.33, *P* < 0.1) (Table 5). Subsequently, discriminatory analysis and ROC curves were used to establish and assess a predictive diagnostic model of CAP with these two independent factors. The predictive diagnostic model of *M. pneumoniae* CAP included the characteristics of being younger than 45 years of age and not having a coexisting disease, with an AUC of 0.61 (95% CI: 0.53 to 0.69). The sensitivity and specificity of this model were 54.9% (95% CI: 51.2% to 64.8%) and 58.0% (95% CI: 43.4% to 66.5%), respectively (*P* < 0.05).

## Discussion

In this study, at least one pathogen was found in 54.2% of the patients, and viruses accounted for most of the cases (36.4%). These results are consistent with previous studies reporting that etiological evidence can be obtained for about half of adult CAP cases, with viruses being associated with about a third [14-16]. Available large-scale epidemiological investigations of adult viral CAP have mainly been conducted in developed countries. In China, Cao *et al.*[17] found that the most common pathogen was *M. pneumoniae* (29.4%), and respiratory viruses were the second most prevalent (9.6%) in 197 outpatients with CAP. In this study, with a larger sample size, we found a greater proportion of virus-associated CAP. Previously, the incidence of CAP due to atypical pathogens from 4,337 patients worldwide between September 1996 to April 2004 was found to be 22% [18]. Another multicenter study on pathogenic agents in 665 adult patients with CAP in China between December 2003 to November 2004 showed that *M. pneumoniae* was the most common type of pathogen (20.7%) [19]. Bao *et al.* also found *M. pneumoniae* as the top etiological pathogens among 402 fever outpatients with CAP in China from January 2007 to January 2008 [20]. In our study, *M. pneumoniae* was the second most prevalent etiologic agent at 18%, indicating that attention should still be paid to *M. pneumoniae* infections in adult CAP outpatients in China. In the last decade, changes in the prevalence of etiological agents of CAP have been observed, such as the decreasing incidence of bacterial CAP and increasing trends in atypical respiratory pathogens and respiratory viruses [21,22]. These changes may be accounted in part by the emergence of PCR technology, either in single or multiplex format, which has greatly improved the sensitivity of diagnostic tests for respiratory viruses (e.g., influenza virus, parainfluenza virus, adenovirus), especially for those that are hard to culture, such as rhinoviruses, coronaviruses and metapneumoviruses [23-25]. Another explanation for the observed changes in CAP-related pathogens is that the use of oral antibiotics by patients at the beginning of the febrile episode can significantly reduce the sensitivity of culture [16,26]. As only about 11% of patients with CAP will usually have positive blood cultures, which are more commonly associated with severe illness [27], it is difficult to obtain a positive blood culture in mild to moderate cases of CAP. In our study, two-thirds of the study patients were previously treated with antibiotics, which significantly limited the sensitivity and specificity of detection.

Mixed infections have been reported in many previous studies [28-30]. In our current investigation, 13.4% of study patients had mixed infections. However, interactions between different pathogens *in vivo* are poorly understood. It is unclear whether a virus alone causes pneumonia or acts in conjunction with other respiratory pathogens, and a favored hypothesis is that a viral infection is followed by a secondary bacterial infection [9,31-33].

In this study, we detected 15 types of respiratory viruses and analyzed their distribution to supplement the epidemiological investigations of adult viral CAP in China. Consistent with previous studies [9,30], we have shown that Flu A was the most commonly detected virus associated with CAP and have drawn attention to this particular pathogen as a cause of pneumonia, especially in the winter. Rhinovirus infections, which are usually limited to the upper respiratory tract but also can cause pneumonia [34], ranked second. The virus distribution in our study was similar to that in previous reports [35-37].

A major current challenge for determining etiological pathogens of adult CAP centers on making the diagnosis based on patient clinical features [38]. In our study, by comparison of monoinfections, we found that *M. pneumoniae* CAP patients were significantly younger than viral or bacterial CAP patients. Cough with expectoration was more common in bacterial CAP patients compared with the other two groups, and dyspnea was significantly different between individuals with viral and bacterial CAP. Similar conclusions have been made in previous reports, yet the results differed among different study populations [39,40]. Therefore, exploring etiological diagnostic models based on combinations of clinical factors in order to identify the causative pathogens has been a focus of CAP research. While diagnostic models of bacterial CAP have been established [41-43], data for such predictive models for viral and *M. pneumoniae* CAP are still lacking. In our study, we attempted to build viral and *M. pneumoniae* diagnostic models based on combinations of clinical characteristics from a study population in China. Factors for the predictive etiological diagnostic model of viral CAP consisted of cough, dyspnea, mild chest pain and WBC counts (4.0 -10.0) × 10^9^/L, and that for *M. pneumoniae* CAP included being younger than 45years old and the absence of a coexisting disease. Corresponding predictors for each model have been reported in the literature. For example, Yang *et al.*[44] described the association of cough with Flu A infection. Johnstone *et al.*[40] reported that a viral infection was usually accompanied by the absence of chest pain and a normal leukocyte count. Ma *et al.* found that independent predictors of viral pneumonia included nursing home residence and absence of leukocytosis [45]. Meanwhile, Cao *et al.*[17] reported that *M. pneumoniae* infection was most common in young pneumonia patients without a coexisting disease. In our study, we combined these clinical characteristics to build the diagnostic models. From the AUC of these models, we can see that with the use of clinical characteristics alone, it would be difficult to determine the causative agent(s) accurately, but they can provide a preliminary etiological diagnosis for CAP patients before laboratory results are available. As the accuracy of CAP etiological diagnostic models may be dependent on treatment type (inpatient or outpatient), age and the number of patient samples [41-43], the specificity and sensitivity of such models need to be further studied. Currently, etiological diagnosis of CAP still depends on laboratory pathogen test results.

While the data obtained was informative, this study had several limitations. One was that we did not investigate the pathogen prevalence in asymptomatic adults from the same population. However, Lieberman *et al.* had previously found a significantly lower proportion of respiratory viruses in asymptomatic control subjects (7.1%) than in CAP patients [31.7% with at least one respiratory virus, including influenza virus (4.4% vs. 0.4% in control) and rhinovirus (4.9% vs. 2.0% in control] [15]. Second, we used different assays (multiplex PCR, quantitative real-time fluorescence PCR, culture and *S. pneumoniae* urinary antigen immunoassay) for different pathogens but did not compare the differences in sensitivity and specificity between these microbiological techniques. Third, two-thirds of study patients self-medicated with antibiotics, which significantly limited the sensitivity and specificity of bacterial detection assays. Fourth, microbiological analysis was not performed for certain respiratory pathogens, such as *Chlamydia pneumoniae*, *Legionella pneumophila* and *Mycobacterium tuberculosis*. Fifth, as the individuals in this population were not severely ill, the findings of this study may not be generalized to hospitalized or severely ill CAP patients. Sixth, the data collection was limited to one year, and the number of study patients may have been insufficient to draw firm conclusions. Therefore, further studies with a larger sample size will be needed to confirm and extend our findings.

## Conclusions

In this study, the distribution of CAP-associated pathogens in adults was consistent with trends from other etiological studies showing that bacterial CAP is decreasing, while CAP-associated respiratory viruses and *M. pneumoniae* are increasing. The results indicate that potential viral and *M. pneumoniae* infections should be given more attention in adult CAP outpatients. Our survey of 15 types of respiratory viruses and analysis of the distribution viruses supplement the epidemiological investigations of adult viral CAP in China. Among the virus strains detected, Flu A virus was found to be the most prevalent from November 2010 to March 2011, emphasizing the importance of this virus in the winter. As identifying the potential causative pathogens of CAP is of major clinical value, we also made progress on building viral and *M. pneumoniae* diagnostic models based on combinations of clinical characteristics. These two models revealed that it would be difficult to arrive at the diagnosis accurately using clinical characteristics alone, but they can provide a preliminary indication of the etiological pathogen(s) before laboratory results become available. At the same time, our results highlight the fact that it is necessary to conduct pathological examinations not only for bacteria but also for viruses and *M. pneumoniae* for etiological diagnosis of CAP patients.

## Abbreviations

CAP: Community-acquired pneumonia; Flu A: Influenza A virus; Flu B: Influenza B virus; PIV: Parainfluenza virus; RSV: Respiratory syncytial virus; AdV: Adenovirus; CoV: Human coronavirus; hRV: Rhinovirus; hBoV: Human bocavirus; hMPV: Human metapneumovirus; EnV: Enterovirus; ROC curves: Receiver Operating Characteristic curves; cDNA: Complementary DNA; PCR: Polymerase chain reaction; WBC: White blood cell counts.

## Competing interest

The authors’ declare that they have no competing interests.

## Authors’ contributions

YFL participated in the experiments, analyzed the data and drafted the initial manuscript. YG conceived and designed the study, helped to analyze the data and modified the manuscript. MFC supervised enrollment and data collection. BC helped to design the study, coordinated the experiments and contributed reagents and materials. XHY participated in enrollment and data collection and helped to analyze the data. LW participated in the design and coordination of the study. All authors have read and approved the final manuscript.

## Pre-publication history

The pre-publication history for this paper can be accessed here:

http://www.biomedcentral.com/1471-2334/13/309/prepub
